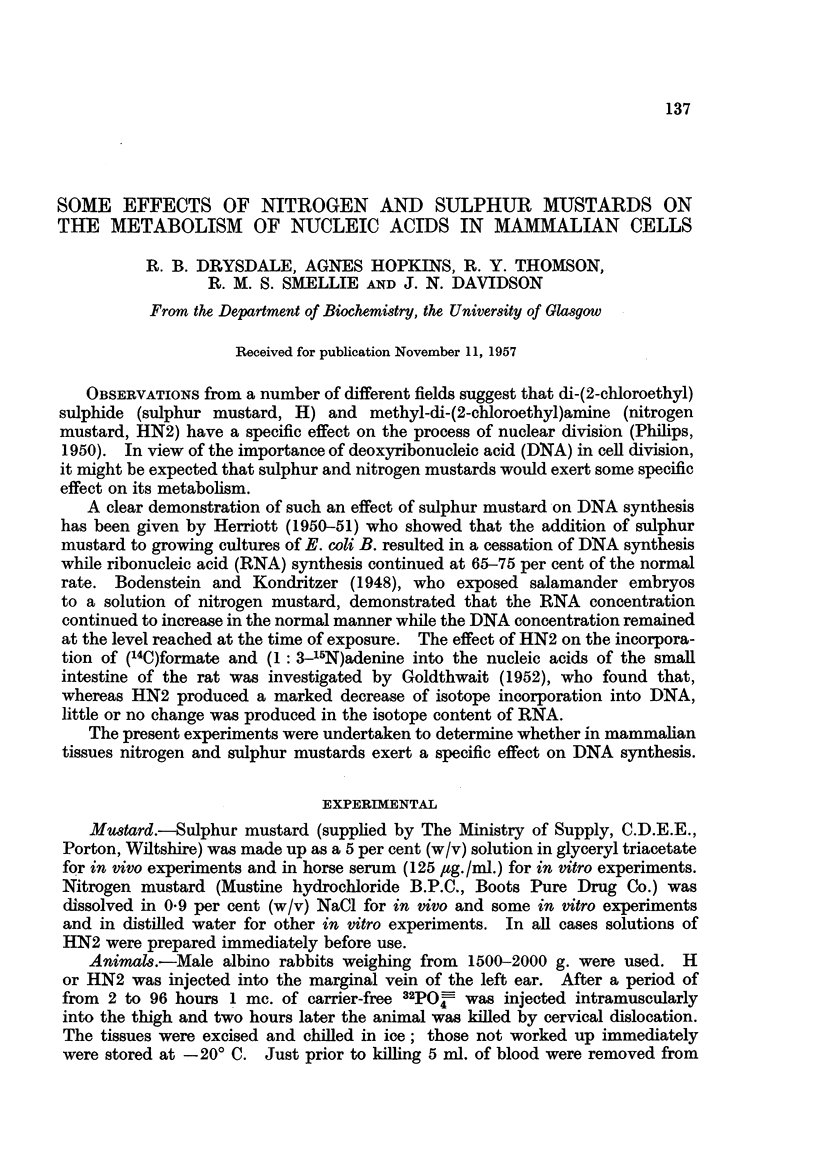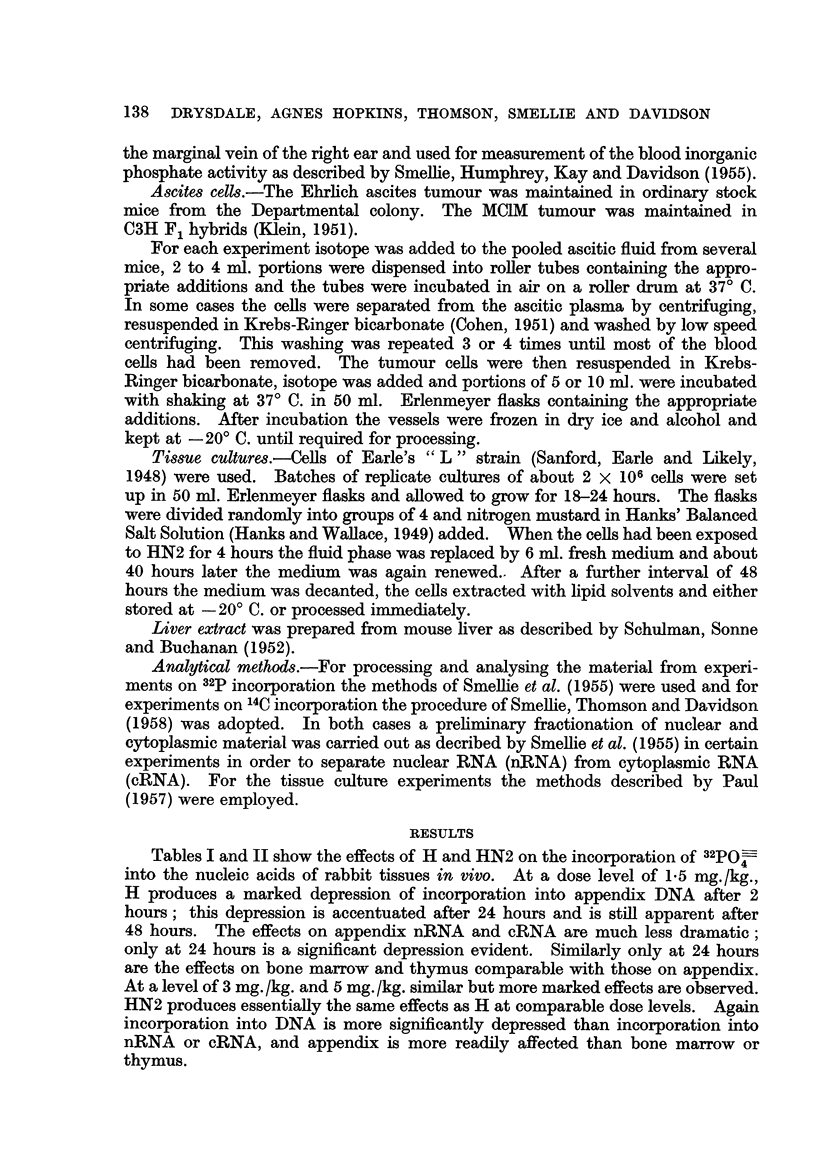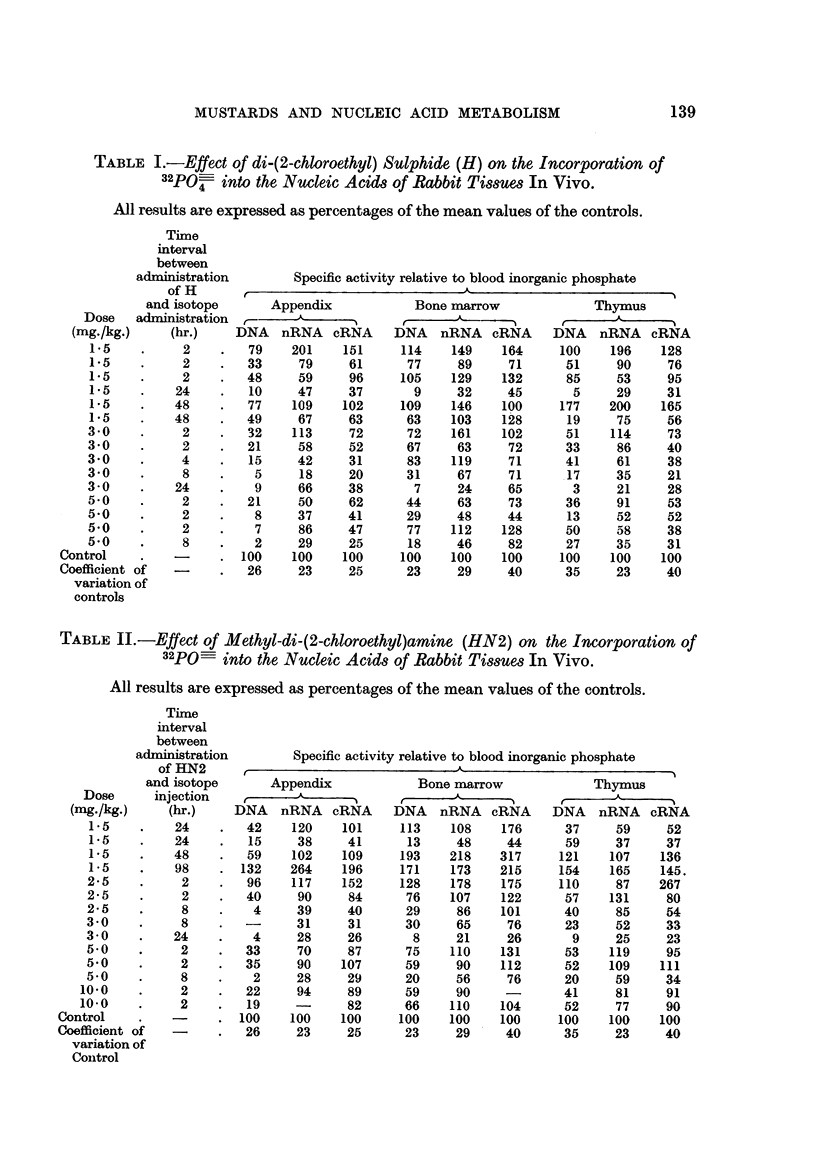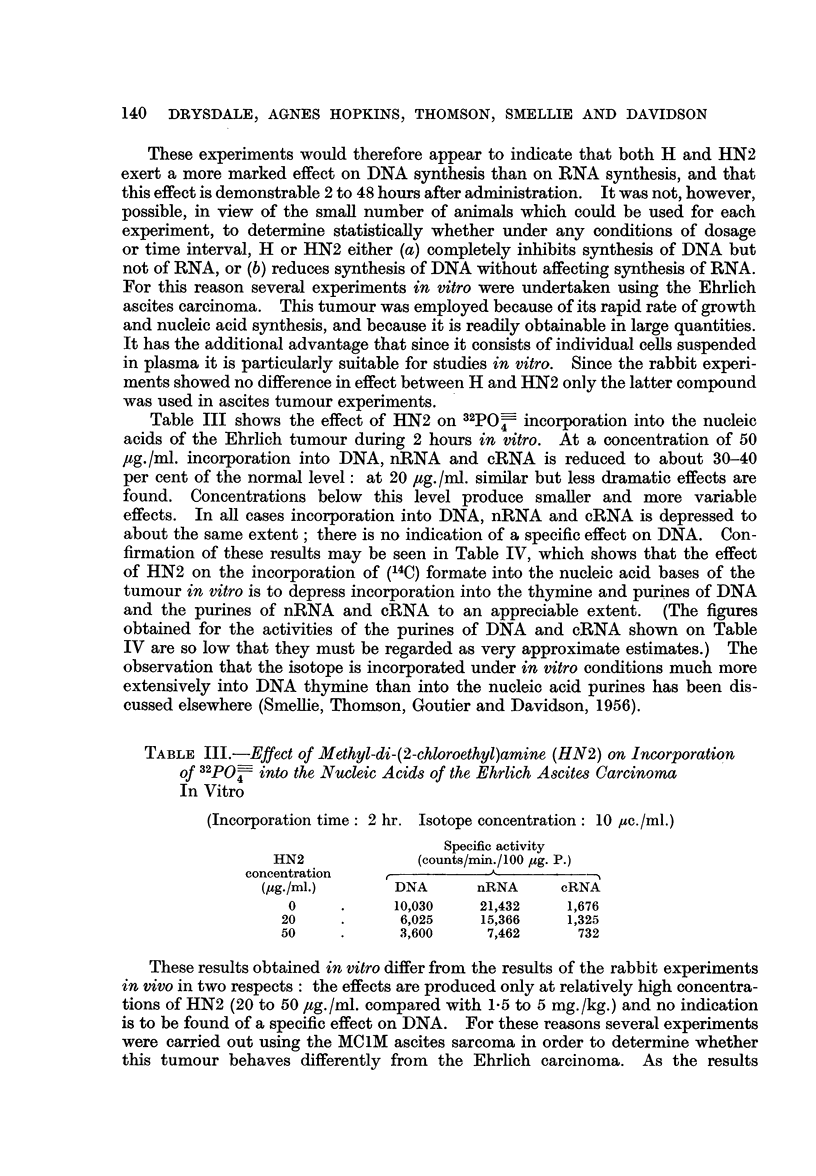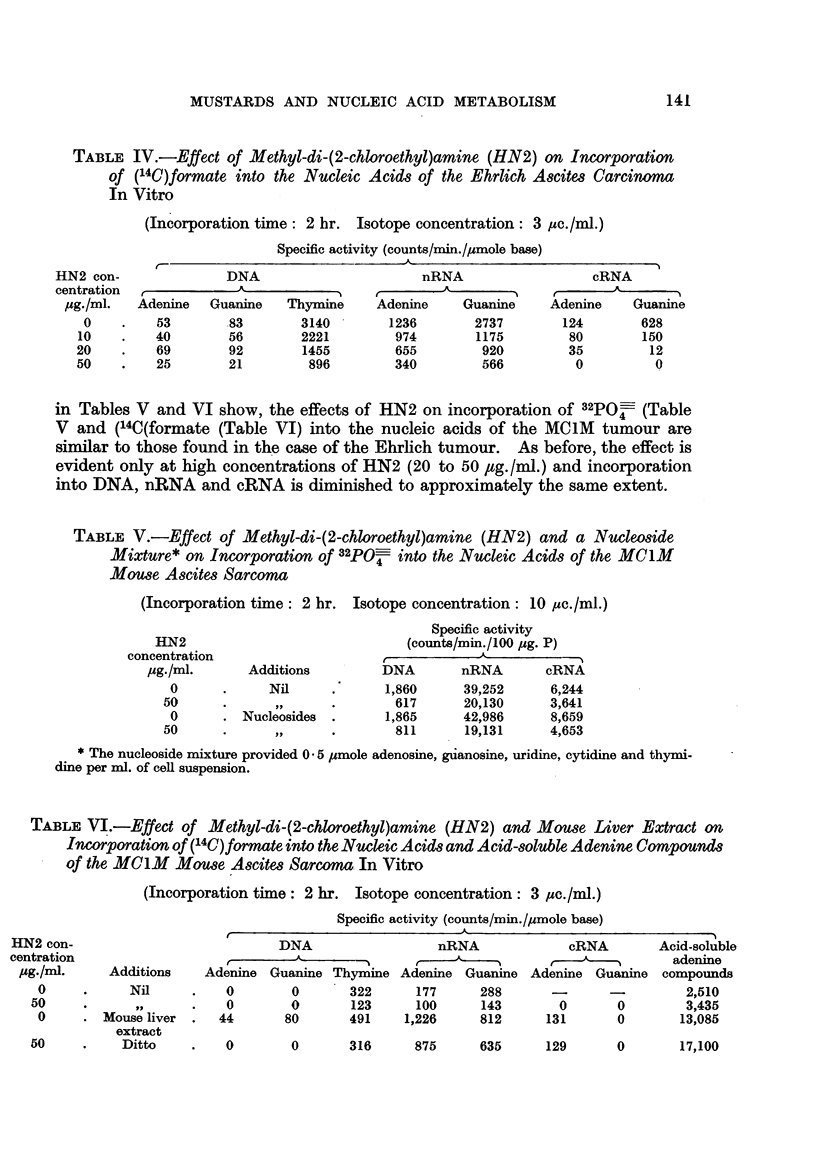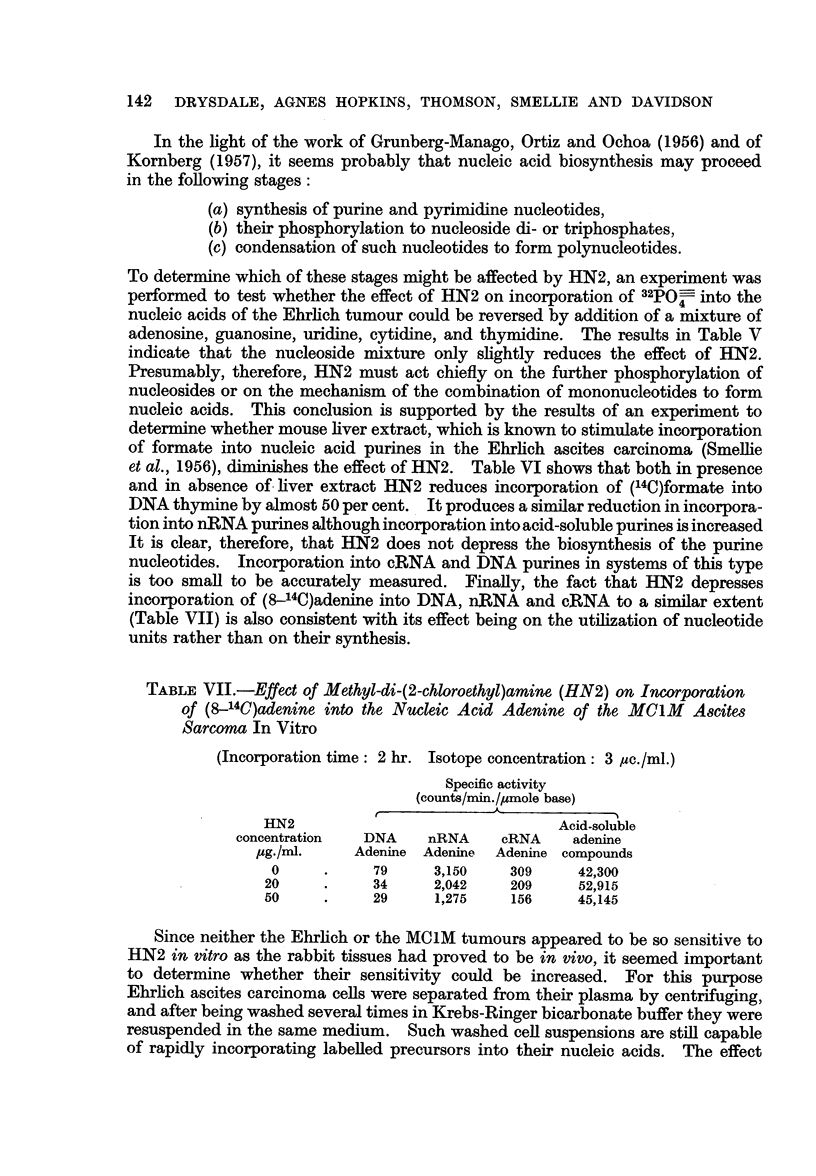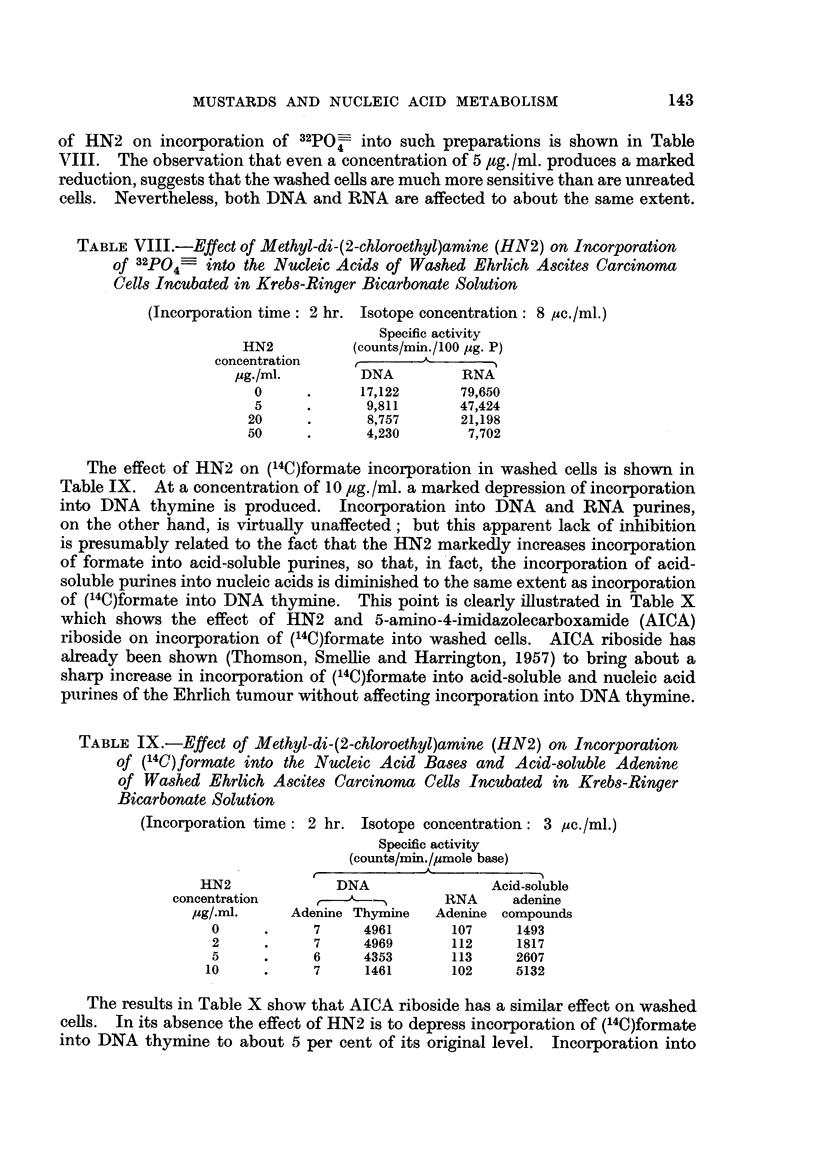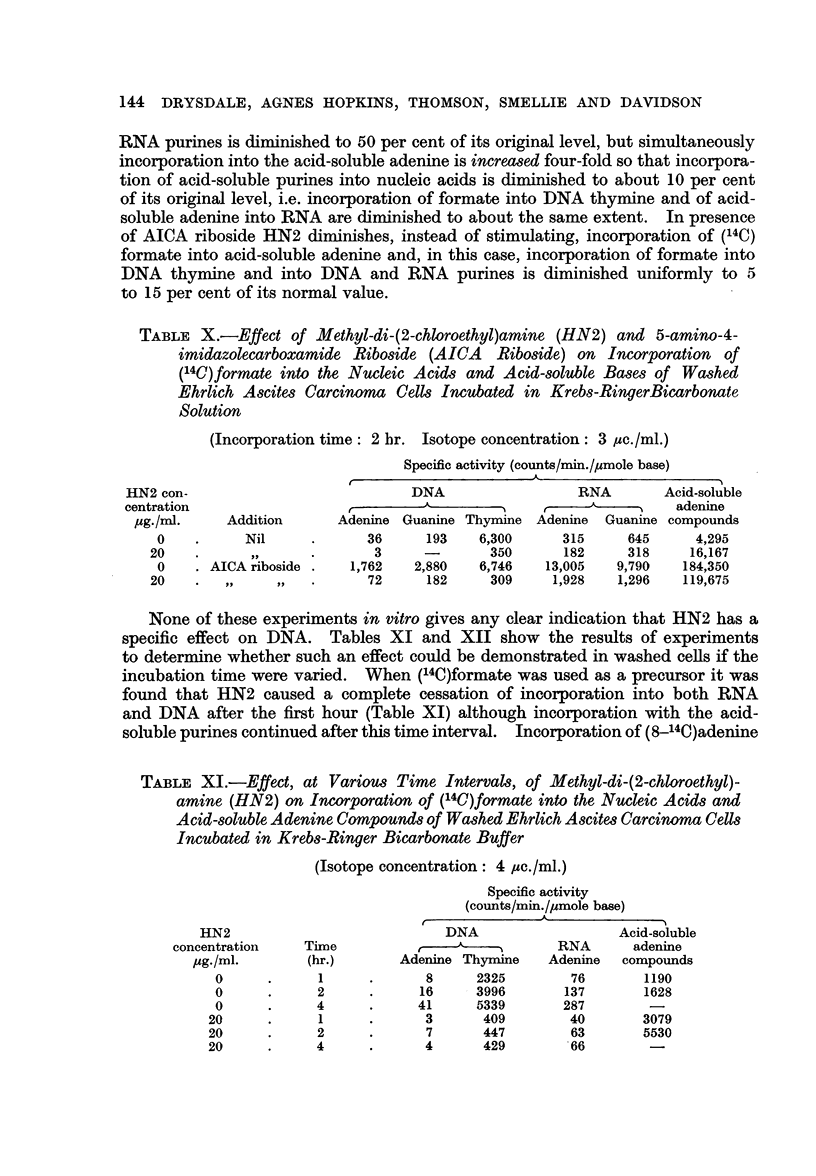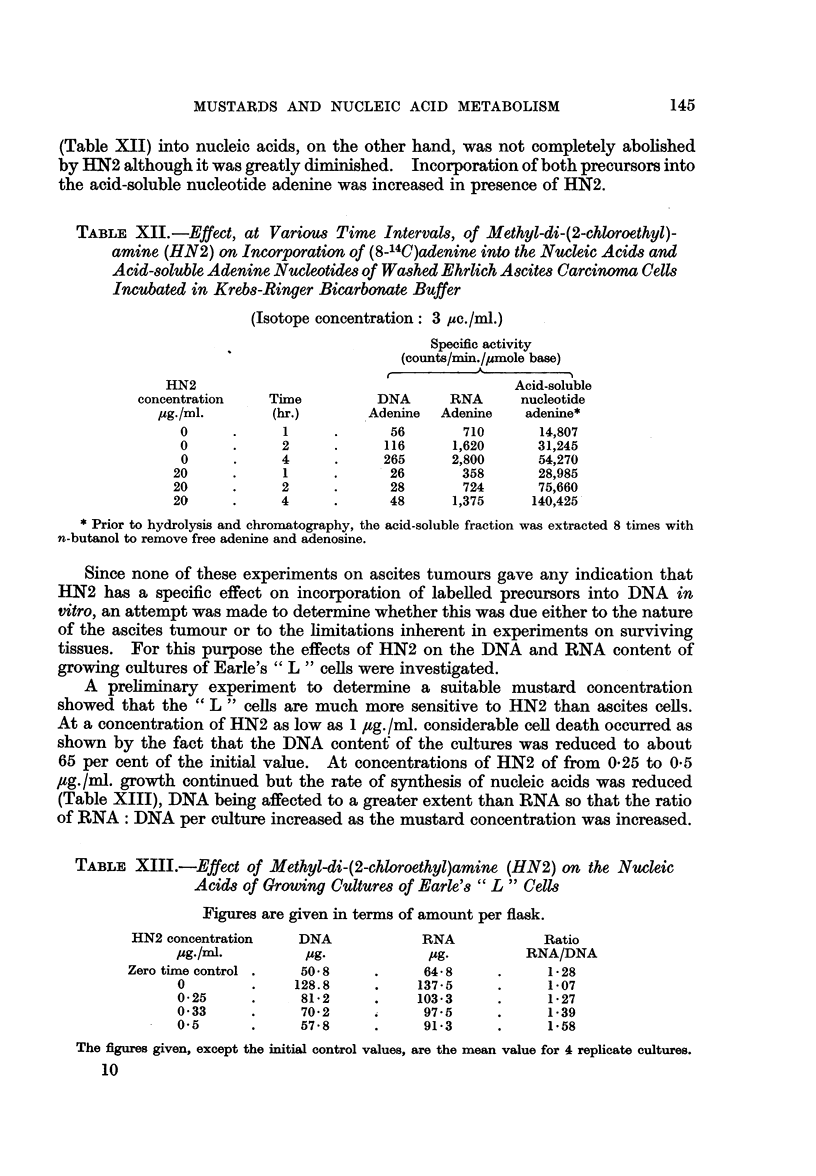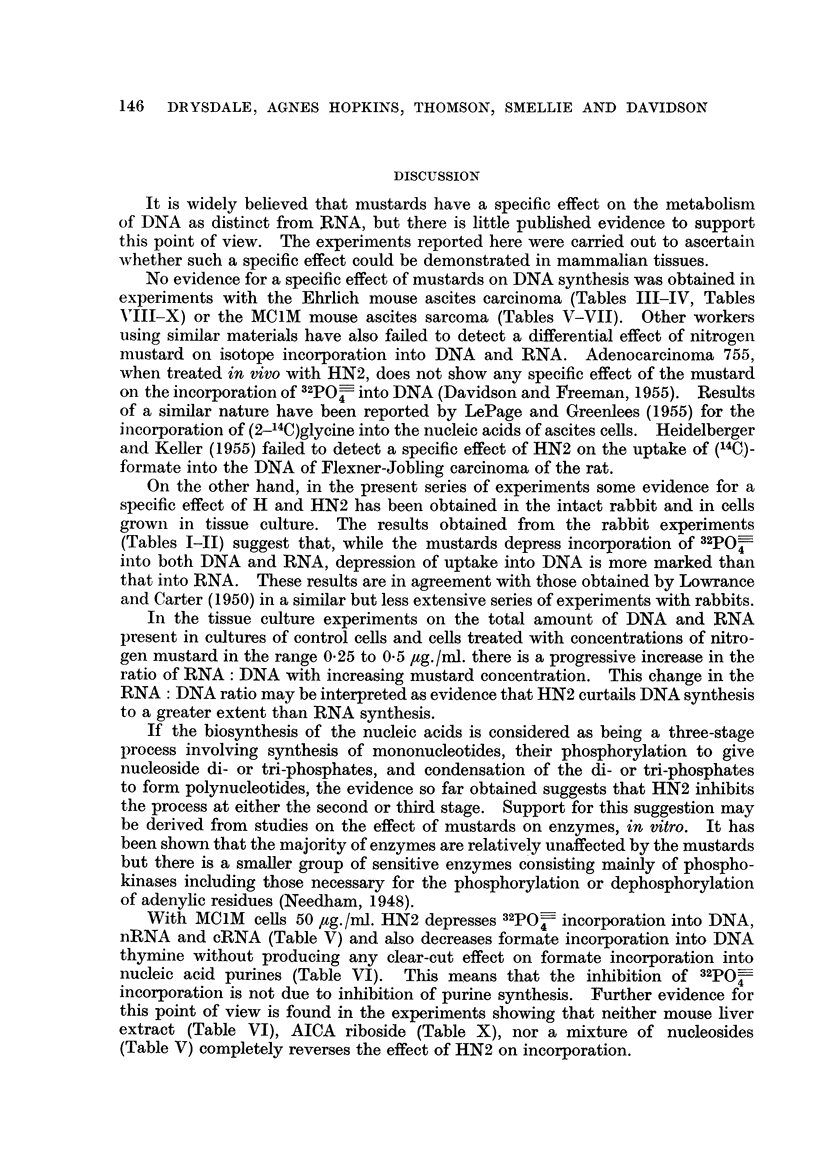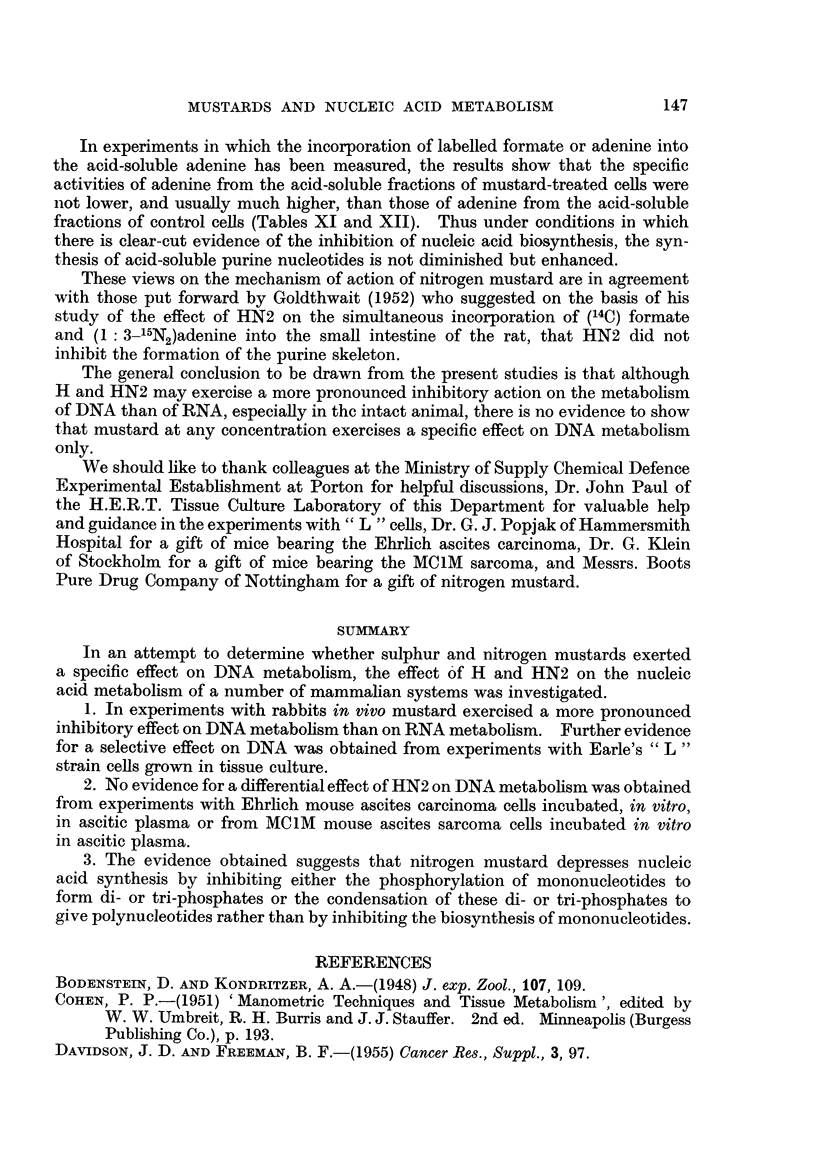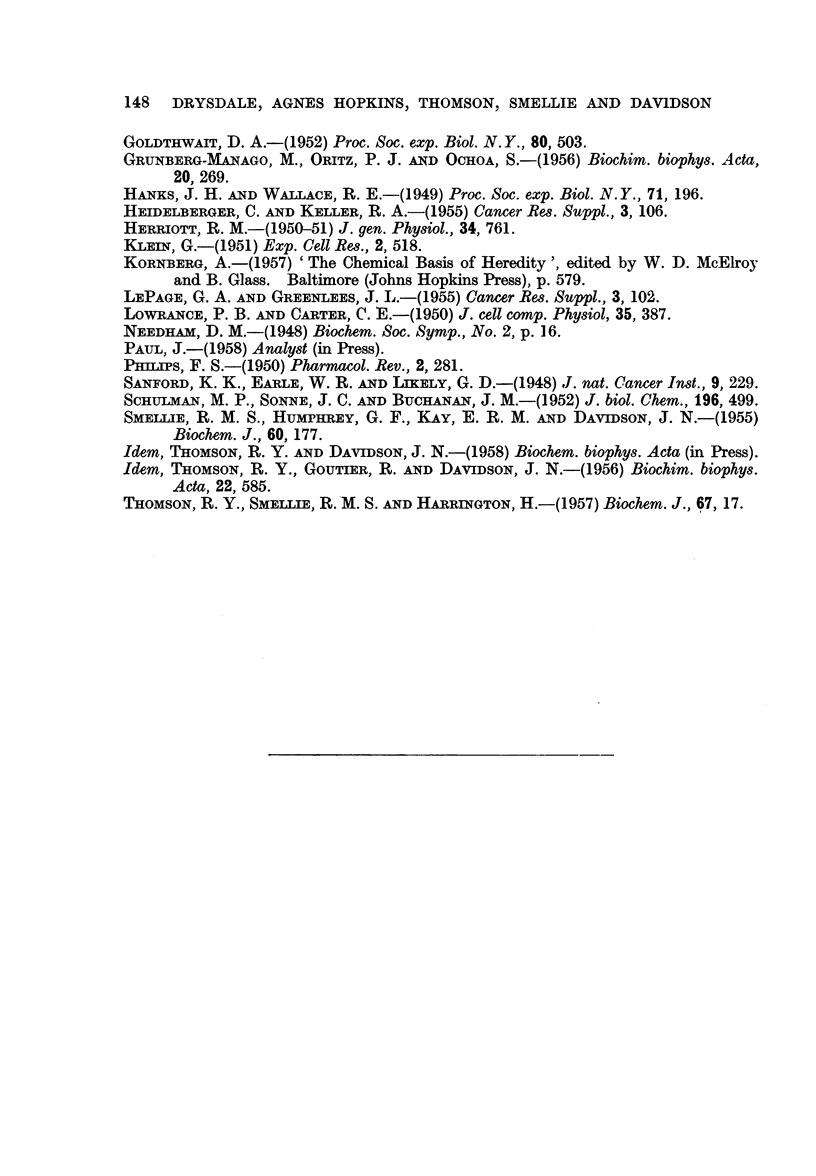# Some Effects of Nitrogen and Sulphur Mustards on the Metabolism of Nucleic Acids in Mammalian Cells

**DOI:** 10.1038/bjc.1958.18

**Published:** 1958-03

**Authors:** R. B. Drysdale, Agnes Hopkins, R. Y. Thomson, R. M. S. Smellie, J. N. Davidson


### SOME EFFECTS OF NITROGEN AND SULPHUR MUSTARDS ON THE METABOLISM OF NUCLEIC ACIDS IN MAMMALIAN CELLS

R. B. DRYSDALE, AGNES HOPKINS, R. Y. THOMSON, R. M. S. SMELLIE AND J. N. DAVIDSON

*From the Department of Biochemistry, the University of Glasgow*

Received for publication November 11, 1957

OBSERVATIONS from a number of different fields suggest that di-(2-chloroethyl) sulphide (sulphur mustard, H) and methyl-di-(2-chloroethyl)amine (nitrogen mustard, HN2) have a specific effect on the process of nuclear division (Philips, 1950). In view of the importance of deoxyribonucleic acid (DNA) in cell division, it might be expected that sulphur and nitrogen mustards would exert some specific effect on its metabolism.

A clear demonstration of such an effect of sulphur mustard on DNA synthesis has been given by Herriott (1950–51) who showed that the addition of sulphur mustard to growing cultures of *E. coli B.* resulted in a cessation of DNA synthesis while ribonucleic acid (RNA) synthesis continued at 65–75 per cent of the normal rate. Bodenstein and Kondritzer (1948), who exposed salamander embryos to a solution of nitrogen mustard, demonstrated that the RNA concentration continued to increase in the normal manner while the DNA concentration remained at the level reached at the time of exposure. The effect of HN2 on the incorporation of ($^{14}$C)formate and (1 : 3–$^{15}$N)adenine into the nucleic acids of the small intestine of the rat was investigated by Goldthwait (1952), who found that, whereas HN2 produced a marked decrease of isotope incorporation into DNA, little or no change was produced in the isotope content of RNA.

The present experiments were undertaken to determine whether in mammalian tissues nitrogen and sulphur mustards exert a specific effect on DNA synthesis.

### EXPERIMENTAL

*Mustard.*—Sulphur mustard (supplied by The Ministry of Supply, C.D.E.E., Porton, Wiltshire) was made up as a 5 per cent (w/v) solution in glyceryl triacetate for *in vivo* experiments and in horse serum (125 $\mu$g./ml.) for *in vitro* experiments. Nitrogen mustard (Mustine hydrochloride B.P.C., Boots Pure Drug Co.) was dissolved in 0·9 per cent (w/v) NaCl for *in vivo* and some *in vitro* experiments and in distilled water for other *in vitro* experiments. In all cases solutions of HN2 were prepared immediately before use.

*Animals.*—Male albino rabbits weighing from 1500–2000 g. were used. H or HN2 was injected into the marginal vein of the left ear. After a period of from 2 to 96 hours 1 mc. of carrier-free $^{32}PO_4^{\equiv}$ was injected intramuscularly into the thigh and two hours later the animal was killed by cervical dislocation. The tissues were excised and chilled in ice ; those not worked up immediately were stored at $-20°$ C. Just prior to killing 5 ml. of blood were removed from the marginal vein of the right ear and used for measurement of the blood inorganic phosphate activity as described by Smellie, Humphrey, Kay and Davidson (1955).

*Ascites cells.*—The Ehrlich ascites tumour was maintained in ordinary stock mice from the Departmental colony. The MClM tumour was maintained in C3H $F_1$ hybrids (Klein, 1951).

For each experiment isotope was added to the pooled ascitic fluid from several mice, 2 to 4 ml. portions were dispensed into roller tubes containing the appropriate additions and the tubes were incubated in air on a roller drum at 37° C. In some cases the cells were separated from the ascitic plasma by centrifuging, resuspended in Krebs-Ringer bicarbonate (Cohen, 1951) and washed by low speed centrifuging. This washing was repeated 3 or 4 times until most of the blood cells had been removed. The tumour cells were then resuspended in Krebs-Ringer bicarbonate, isotope was added and portions of 5 or 10 ml. were incubated with shaking at 37° C. in 50 ml. Erlenmeyer flasks containing the appropriate additions. After incubation the vessels were frozen in dry ice and alcohol and kept at −20° C. until required for processing.

*Tissue cultures.*—Cells of Earle's " L " strain (Sanford, Earle and Likely, 1948) were used. Batches of replicate cultures of about $2 \times 10^6$ cells were set up in 50 ml. Erlenmeyer flasks and allowed to grow for 18–24 hours. The flasks were divided randomly into groups of 4 and nitrogen mustard in Hanks' Balanced Salt Solution (Hanks and Wallace, 1949) added. When the cells had been exposed to HN2 for 4 hours the fluid phase was replaced by 6 ml. fresh medium and about 40 hours later the medium was again renewed. After a further interval of 48 hours the medium was decanted, the cells extracted with lipid solvents and either stored at −20° C. or processed immediately.

*Liver extract* was prepared from mouse liver as described by Schulman, Sonne and Buchanan (1952).

*Analytical methods.*—For processing and analysing the material from experiments on $^{32}$P incorporation the methods of Smellie *et al.* (1955) were used and for experiments on $^{14}$C incorporation the procedure of Smellie, Thomson and Davidson (1958) was adopted. In both cases a preliminary fractionation of nuclear and cytoplasmic material was carried out as decribed by Smellie *et al.* (1955) in certain experiments in order to separate nuclear RNA (nRNA) from cytoplasmic RNA (cRNA). For the tissue culture experiments the methods described by Paul (1957) were employed.

### RESULTS

Tables I and II show the effects of H and HN2 on the incorporation of $^{32}PO_4^{\equiv}$ into the nucleic acids of rabbit tissues *in vivo*. At a dose level of 1·5 mg./kg., H produces a marked depression of incorporation into appendix DNA after 2 hours ; this depression is accentuated after 24 hours and is still apparent after 48 hours. The effects on appendix nRNA and cRNA are much less dramatic ; only at 24 hours is a significant depression evident. Similarly only at 24 hours are the effects on bone marrow and thymus comparable with those on appendix. At a level of 3 mg./kg. and 5 mg./kg. similar but more marked effects are observed. HN2 produces essentially the same effects as H at comparable dose levels. Again incorporation into DNA is more significantly depressed than incorporation into nRNA or cRNA, and appendix is more readily affected than bone marrow or thymus.

TABLE I.—*Effect of di-(2-chloroethyl) Sulphide (H) on the Incorporation of* $^{32}PO_4^{\equiv}$ *into the Nucleic Acids of Rabbit Tissues* In Vivo.

All results are expressed as percentages of the mean values of the controls.

| Dose (mg./kg.) | Time interval between administration of H and isotope administration (hr.) | Specific activity relative to blood inorganic phosphate: Appendix DNA | Appendix nRNA | Appendix cRNA | Bone marrow DNA | Bone marrow nRNA | Bone marrow cRNA | Thymus DNA | Thymus nRNA | Thymus cRNA |
|---|---|---|---|---|---|---|---|---|---|---|
| 1·5 | 2 | 79 | 201 | 151 | 114 | 149 | 164 | 100 | 196 | 128 |
| 1·5 | 2 | 33 | 79 | 61 | 77 | 89 | 71 | 51 | 90 | 76 |
| 1·5 | 2 | 48 | 59 | 96 | 105 | 129 | 132 | 85 | 53 | 95 |
| 1·5 | 24 | 10 | 47 | 37 | 9 | 32 | 45 | 5 | 29 | 31 |
| 1·5 | 48 | 77 | 109 | 102 | 109 | 146 | 100 | 177 | 200 | 165 |
| 1·5 | 48 | 49 | 67 | 63 | 63 | 103 | 128 | 19 | 75 | 56 |
| 3·0 | 2 | 32 | 113 | 72 | 72 | 161 | 102 | 51 | 114 | 73 |
| 3·0 | 2 | 21 | 58 | 52 | 67 | 63 | 72 | 33 | 86 | 40 |
| 3·0 | 4 | 15 | 42 | 31 | 83 | 119 | 71 | 41 | 61 | 38 |
| 3·0 | 8 | 5 | 18 | 20 | 31 | 67 | 71 | 17 | 35 | 21 |
| 3·0 | 24 | 9 | 66 | 38 | 7 | 24 | 65 | 3 | 21 | 28 |
| 5·0 | 2 | 21 | 50 | 62 | 44 | 63 | 73 | 36 | 91 | 53 |
| 5·0 | 2 | 8 | 37 | 41 | 29 | 48 | 44 | 13 | 52 | 52 |
| 5·0 | 2 | 7 | 86 | 47 | 77 | 112 | 128 | 50 | 58 | 38 |
| 5·0 | 8 | 2 | 29 | 25 | 18 | 46 | 82 | 27 | 35 | 31 |
| Control | — | 100 | 100 | 100 | 100 | 100 | 100 | 100 | 100 | 100 |
| Coefficient of variation of controls | — | 26 | 23 | 25 | 23 | 29 | 40 | 35 | 23 | 40 |

TABLE II.—*Effect of Methyl-di-(2-chloroethyl)amine (HN2) on the Incorporation of* $^{32}PO^{\equiv}$ *into the Nucleic Acids of Rabbit Tissues* In Vivo.

All results are expressed as percentages of the mean values of the controls.

| Dose (mg./kg.) | Time interval between administration of HN2 and isotope injection (hr.) | Specific activity relative to blood inorganic phosphate: Appendix DNA | Appendix nRNA | Appendix cRNA | Bone marrow DNA | Bone marrow nRNA | Bone marrow cRNA | Thymus DNA | Thymus nRNA | Thymus cRNA |
|---|---|---|---|---|---|---|---|---|---|---|
| 1·5 | 24 | 42 | 120 | 101 | 113 | 108 | 176 | 37 | 59 | 52 |
| 1·5 | 24 | 15 | 38 | 41 | 13 | 48 | 44 | 59 | 37 | 37 |
| 1·5 | 48 | 59 | 102 | 109 | 193 | 218 | 317 | 121 | 107 | 136 |
| 1·5 | 98 | 132 | 264 | 196 | 171 | 173 | 215 | 154 | 165 | 145. |
| 2·5 | 2 | 96 | 117 | 152 | 128 | 178 | 175 | 110 | 87 | 267 |
| 2·5 | 2 | 40 | 90 | 84 | 76 | 107 | 122 | 57 | 131 | 80 |
| 2·5 | 8 | 4 | 39 | 40 | 29 | 86 | 101 | 40 | 85 | 54 |
| 3·0 | 8 | — | 31 | 31 | 30 | 65 | 76 | 23 | 52 | 33 |
| 3·0 | 24 | 4 | 28 | 26 | 8 | 21 | 26 | 9 | 25 | 23 |
| 5·0 | 2 | 33 | 70 | 87 | 75 | 110 | 131 | 53 | 119 | 95 |
| 5·0 | 2 | 35 | 90 | 107 | 59 | 90 | 112 | 52 | 109 | 111 |
| 5·0 | 8 | 2 | 28 | 29 | 20 | 56 | 76 | 20 | 59 | 34 |
| 10·0 | 2 | 22 | 94 | 89 | 59 | 90 | — | 41 | 81 | 91 |
| 10·0 | 2 | 19 | — | 82 | 66 | 110 | 104 | 52 | 77 | 90 |
| Control | — | 100 | 100 | 100 | 100 | 100 | 100 | 100 | 100 | 100 |
| Coefficient of variation of Control | — | 26 | 23 | 25 | 23 | 29 | 40 | 35 | 23 | 40 |

These experiments would therefore appear to indicate that both H and HN2 exert a more marked effect on DNA synthesis than on RNA synthesis, and that this effect is demonstrable 2 to 48 hours after administration. It was not, however, possible, in view of the small number of animals which could be used for each experiment, to determine statistically whether under any conditions of dosage or time interval, H or HN2 either (*a*) completely inhibits synthesis of DNA but not of RNA, or (*b*) reduces synthesis of DNA without affecting synthesis of RNA. For this reason several experiments *in vitro* were undertaken using the Ehrlich ascites carcinoma. This tumour was employed because of its rapid rate of growth and nucleic acid synthesis, and because it is readily obtainable in large quantities. It has the additional advantage that since it consists of individual cells suspended in plasma it is particularly suitable for studies *in vitro.* Since the rabbit experiments showed no difference in effect between H and HN2 only the latter compound was used in ascites tumour experiments.

Table III shows the effect of HN2 on $^{32}PO_4^{\equiv}$ incorporation into the nucleic acids of the Ehrlich tumour during 2 hours *in vitro.* At a concentration of 50 $\mu$g./ml. incorporation into DNA, nRNA and cRNA is reduced to about 30–40 per cent of the normal level : at 20 $\mu$g./ml. similar but less dramatic effects are found. Concentrations below this level produce smaller and more variable effects. In all cases incorporation into DNA, nRNA and cRNA is depressed to about the same extent ; there is no indication of a specific effect on DNA. Confirmation of these results may be seen in Table IV, which shows that the effect of HN2 on the incorporation of ($^{14}$C) formate into the nucleic acid bases of the tumour *in vitro* is to depress incorporation into the thymine and purines of DNA and the purines of nRNA and cRNA to an appreciable extent. (The figures obtained for the activities of the purines of DNA and cRNA shown on Table IV are so low that they must be regarded as very approximate estimates.) The observation that the isotope is incorporated under *in vitro* conditions much more extensively into DNA thymine than into the nucleic acid purines has been discussed elsewhere (Smellie, Thomson, Goutier and Davidson, 1956).

TABLE III.—*Effect of Methyl-di-(2-chloroethyl)amine (HN2) on Incorporation of $^{32}PO_4^{\equiv}$ into the Nucleic Acids of the Ehrlich Ascites Carcinoma* In Vitro

(Incorporation time : 2 hr. Isotope concentration : 10 $\mu$c./ml.)

| HN2 concentration ($\mu$g./ml.) | Specific activity (counts/min./100 $\mu$g. P.) | | |
|---|---|---|---|
| | DNA | nRNA | cRNA |
| 0 | 10,030 | 21,432 | 1,676 |
| 20 | 6,025 | 15,366 | 1,325 |
| 50 | 3,600 | 7,462 | 732 |

These results obtained *in vitro* differ from the results of the rabbit experiments *in vivo* in two respects : the effects are produced only at relatively high concentrations of HN2 (20 to 50 $\mu$g./ml. compared with 1·5 to 5 mg./kg.) and no indication is to be found of a specific effect on DNA. For these reasons several experiments were carried out using the MC1M ascites sarcoma in order to determine whether this tumour behaves differently from the Ehrlich carcinoma. As the results

TABLE IV.—*Effect of Methyl-di-(2-chloroethyl)amine (HN2) on Incorporation of ($^{14}$C)formate into the Nucleic Acids of the Ehrlich Ascites Carcinoma* In Vitro

(Incorporation time : 2 hr. Isotope concentration : 3 μc./ml.)

| HN2 concentration μg./ml. | Specific activity (counts/min./μmole base) DNA | | | nRNA | | cRNA | |
|---|---|---|---|---|---|---|---|
| | Adenine | Guanine | Thymine | Adenine | Guanine | Adenine | Guanine |
| 0 | 53 | 83 | 3140 | 1236 | 2737 | 124 | 628 |
| 10 | 40 | 56 | 2221 | 974 | 1175 | 80 | 150 |
| 20 | 69 | 92 | 1455 | 655 | 920 | 35 | 12 |
| 50 | 25 | 21 | 896 | 340 | 566 | 0 | 0 |

in Tables V and VI show, the effects of HN2 on incorporation of $^{32}PO_4^{\equiv}$ (Table V and ($^{14}$C(formate (Table VI) into the nucleic acids of the MC1M tumour are similar to those found in the case of the Ehrlich tumour. As before, the effect is evident only at high concentrations of HN2 (20 to 50 μg./ml.) and incorporation into DNA, nRNA and cRNA is diminished to approximately the same extent.

TABLE V.—*Effect of Methyl-di-(2-chloroethyl)amine (HN2) and a Nucleoside Mixture* on Incorporation of $^{32}PO_4^{\equiv}$ into the Nucleic Acids of the MC1M Mouse Ascites Sarcoma*

(Incorporation time : 2 hr. Isotope concentration : 10 μc./ml.)

| HN2 concentration μg./ml. | Additions | Specific activity (counts/min./100 μg. P) DNA | nRNA | cRNA |
|---|---|---|---|---|
| 0 | Nil | 1,860 | 39,252 | 6,244 |
| 50 | „ | 617 | 20,130 | 3,641 |
| 0 | Nucleosides | 1,865 | 42,986 | 8,659 |
| 50 | „ | 811 | 19,131 | 4,653 |

* The nucleoside mixture provided 0·5 μmole adenosine, guanosine, uridine, cytidine and thymidine per ml. of cell suspension.

TABLE VI.—*Effect of Methyl-di-(2-chloroethyl)amine (HN2) and Mouse Liver Extract on Incorporation of ($^{14}$C) formate into the Nucleic Acids and Acid-soluble Adenine Compounds of the MC1M Mouse Ascites Sarcoma* In Vitro

(Incorporation time : 2 hr. Isotope concentration : 3 μc./ml.)

| HN2 concentration μg./ml. | Additions | Specific activity (counts/min./μmole base) DNA | | | nRNA | | cRNA | | Acid-soluble adenine compounds |
|---|---|---|---|---|---|---|---|---|---|
| | | Adenine | Guanine | Thymine | Adenine | Guanine | Adenine | Guanine | |
| 0 | Nil | 0 | 0 | 322 | 177 | 288 | — | — | 2,510 |
| 50 | „ | 0 | 0 | 123 | 100 | 143 | 0 | 0 | 3,435 |
| 0 | Mouse liver extract | 44 | 80 | 491 | 1,226 | 812 | 131 | 0 | 13,085 |
| 50 | Ditto | 0 | 0 | 316 | 875 | 635 | 129 | 0 | 17,100 |

In the light of the work of Grunberg-Manago, Ortiz and Ochoa (1956) and of Kornberg (1957), it seems probably that nucleic acid biosynthesis may proceed in the following stages :

(*a*) synthesis of purine and pyrimidine nucleotides,
(*b*) their phosphorylation to nucleoside di- or triphosphates,
(*c*) condensation of such nucleotides to form polynucleotides.

To determine which of these stages might be affected by HN2, an experiment was performed to test whether the effect of HN2 on incorporation of $^{32}PO_4^{\equiv}$ into the nucleic acids of the Ehrlich tumour could be reversed by addition of a mixture of adenosine, guanosine, uridine, cytidine, and thymidine. The results in Table V indicate that the nucleoside mixture only slightly reduces the effect of HN2. Presumably, therefore, HN2 must act chiefly on the further phosphorylation of nucleosides or on the mechanism of the combination of mononucleotides to form nucleic acids. This conclusion is supported by the results of an experiment to determine whether mouse liver extract, which is known to stimulate incorporation of formate into nucleic acid purines in the Ehrlich ascites carcinoma (Smellie *et al.*, 1956), diminishes the effect of HN2. Table VI shows that both in presence and in absence of liver extract HN2 reduces incorporation of ($^{14}$C)formate into DNA thymine by almost 50 per cent. It produces a similar reduction in incorporation into nRNA purines although incorporation into acid-soluble purines is increased It is clear, therefore, that HN2 does not depress the biosynthesis of the purine nucleotides. Incorporation into cRNA and DNA purines in systems of this type is too small to be accurately measured. Finally, the fact that HN2 depresses incorporation of (8–$^{14}$C)adenine into DNA, nRNA and cRNA to a similar extent (Table VII) is also consistent with its effect being on the utilization of nucleotide units rather than on their synthesis.

TABLE VII.—*Effect of Methyl-di-(2-chloroethyl)amine (HN2) on Incorporation of (8–$^{14}$C)adenine into the Nucleic Acid Adenine of the MC1M Ascites Sarcoma* In Vitro

(Incorporation time : 2 hr. Isotope concentration : 3 $\mu$c./ml.)

| HN2 concentration $\mu$g./ml. | Specific activity (counts/min./$\mu$mole base): DNA Adenine | nRNA Adenine | cRNA Adenine | Acid-soluble adenine compounds |
|---|---|---|---|---|
| 0 | 79 | 3,150 | 309 | 42,300 |
| 20 | 34 | 2,042 | 209 | 52,915 |
| 50 | 29 | 1,275 | 156 | 45,145 |

Since neither the Ehrlich or the MC1M tumours appeared to be so sensitive to HN2 *in vitro* as the rabbit tissues had proved to be *in vivo*, it seemed important to determine whether their sensitivity could be increased. For this purpose Ehrlich ascites carcinoma cells were separated from their plasma by centrifuging, and after being washed several times in Krebs-Ringer bicarbonate buffer they were resuspended in the same medium. Such washed cell suspensions are still capable of rapidly incorporating labelled precursors into their nucleic acids. The effect of HN2 on incorporation of $^{32}PO_4^{\equiv}$ into such preparations is shown in Table VIII. The observation that even a concentration of 5 $\mu$g./ml. produces a marked reduction, suggests that the washed cells are much more sensitive than are unreated cells. Nevertheless, both DNA and RNA are affected to about the same extent.

TABLE VIII.—*Effect of Methyl-di-(2-chloroethyl)amine (HN2) on Incorporation of $^{32}PO_4^{\equiv}$ into the Nucleic Acids of Washed Ehrlich Ascites Carcinoma Cells Incubated in Krebs-Ringer Bicarbonate Solution*

(Incorporation time : 2 hr. Isotope concentration : 8 $\mu$c./ml.)

| HN2 concentration $\mu$g./ml. | Specific activity (counts/min./100 $\mu$g. P) DNA | RNA |
|---|---|---|
| 0 | 17,122 | 79,650 |
| 5 | 9,811 | 47,424 |
| 20 | 8,757 | 21,198 |
| 50 | 4,230 | 7,702 |

The effect of HN2 on ($^{14}$C)formate incorporation in washed cells is shown in Table IX. At a concentration of 10 $\mu$g./ml. a marked depression of incorporation into DNA thymine is produced. Incorporation into DNA and RNA purines, on the other hand, is virtually unaffected ; but this apparent lack of inhibition is presumably related to the fact that the HN2 markedly increases incorporation of formate into acid-soluble purines, so that, in fact, the incorporation of acid-soluble purines into nucleic acids is diminished to the same extent as incorporation of ($^{14}$C)formate into DNA thymine. This point is clearly illustrated in Table X which shows the effect of HN2 and 5-amino-4-imidazolecarboxamide (AICA) riboside on incorporation of ($^{14}$C)formate into washed cells. AICA riboside has already been shown (Thomson, Smellie and Harrington, 1957) to bring about a sharp increase in incorporation of ($^{14}$C)formate into acid-soluble and nucleic acid purines of the Ehrlich tumour without affecting incorporation into DNA thymine.

TABLE IX.—*Effect of Methyl-di-(2-chloroethyl)amine (HN2) on Incorporation of ($^{14}$C)formate into the Nucleic Acid Bases and Acid-soluble Adenine of Washed Ehrlich Ascites Carcinoma Cells Incubated in Krebs-Ringer Bicarbonate Solution*

(Incorporation time : 2 hr. Isotope concentration : 3 $\mu$c./ml.)

| HN2 concentration $\mu$g/.ml. | Specific activity (counts/min./$\mu$mole base) DNA Adenine | Thymine | RNA Adenine | Acid-soluble adenine compounds |
|---|---|---|---|---|
| 0 | 7 | 4961 | 107 | 1493 |
| 2 | 7 | 4969 | 112 | 1817 |
| 5 | 6 | 4353 | 113 | 2607 |
| 10 | 7 | 1461 | 102 | 5132 |

The results in Table X show that AICA riboside has a similar effect on washed cells. In its absence the effect of HN2 is to depress incorporation of ($^{14}$C)formate into DNA thymine to about 5 per cent of its original level. Incorporation into

RNA purines is diminished to 50 per cent of its original level, but simultaneously incorporation into the acid-soluble adenine is *increased* four-fold so that incorporation of acid-soluble purines into nucleic acids is diminished to about 10 per cent of its original level, i.e. incorporation of formate into DNA thymine and of acid-soluble adenine into RNA are diminished to about the same extent. In presence of AICA riboside HN2 diminishes, instead of stimulating, incorporation of ($^{14}$C) formate into acid-soluble adenine and, in this case, incorporation of formate into DNA thymine and into DNA and RNA purines is diminished uniformly to 5 to 15 per cent of its normal value.

TABLE X.—*Effect of Methyl-di-(2-chloroethyl)amine (HN2) and 5-amino-4-imidazolecarboxamide Riboside (AICA Riboside) on Incorporation of ($^{14}$C)formate into the Nucleic Acids and Acid-soluble Bases of Washed Ehrlich Ascites Carcinoma Cells Incubated in Krebs-RingerBicarbonate Solution*

(Incorporation time : 2 hr. Isotope concentration : 3 μc./ml.)

| | | Specific activity (counts/min./μmole base) | | | | | |
|---|---|---|---|---|---|---|---|
| | | DNA | | | RNA | | Acid-soluble adenine |
| HN2 concentration μg./ml. | Addition | Adenine | Guanine | Thymine | Adenine | Guanine | compounds |
| 0 | Nil | 36 | 193 | 6,300 | 315 | 645 | 4,295 |
| 20 | „ | 3 | — | 350 | 182 | 318 | 16,167 |
| 0 | AICA riboside | 1,762 | 2,880 | 6,746 | 13,005 | 9,790 | 184,350 |
| 20 | „ „ | 72 | 182 | 309 | 1,928 | 1,296 | 119,675 |

None of these experiments *in vitro* gives any clear indication that HN2 has a specific effect on DNA. Tables XI and XII show the results of experiments to determine whether such an effect could be demonstrated in washed cells if the incubation time were varied. When ($^{14}$C)formate was used as a precursor it was found that HN2 caused a complete cessation of incorporation into both RNA and DNA after the first hour (Table XI) although incorporation with the acid-soluble purines continued after this time interval. Incorporation of (8–$^{14}$C)adenine

TABLE XI.—*Effect, at Various Time Intervals, of Methyl-di-(2-chloroethyl)-amine (HN2) on Incorporation of ($^{14}$C)formate into the Nucleic Acids and Acid-soluble Adenine Compounds of Washed Ehrlich Ascites Carcinoma Cells Incubated in Krebs-Ringer Bicarbonate Buffer*

(Isotope concentration : 4 μc./ml.)

| | | Specific activity (counts/min./μmole base) | | | |
|---|---|---|---|---|---|
| | | DNA | | | Acid-soluble |
| HN2 concentration μg./ml. | Time (hr.) | Adenine | Thymine | RNA Adenine | adenine compounds |
| 0 | 1 | 8 | 2325 | 76 | 1190 |
| 0 | 2 | 16 | 3996 | 137 | 1628 |
| 0 | 4 | 41 | 5339 | 287 | — |
| 20 | 1 | 3 | 409 | 40 | 3079 |
| 20 | 2 | 7 | 447 | 63 | 5530 |
| 20 | 4 | 4 | 429 | 66 | — |

(Table XII) into nucleic acids, on the other hand, was not completely abolished by HN2 although it was greatly diminished. Incorporation of both precursors into the acid-soluble nucleotide adenine was increased in presence of HN2.

TABLE XII.—*Effect, at Various Time Intervals, of Methyl-di-(2-chloroethyl)-amine (HN2) on Incorporation of (8-$^{14}$C)adenine into the Nucleic Acids and Acid-soluble Adenine Nucleotides of Washed Ehrlich Ascites Carcinoma Cells Incubated in Krebs-Ringer Bicarbonate Buffer*

(Isotope concentration: 3 μc./ml.)

| | | Specific activity (counts/min./μmole base) | | |
|---|---|---|---|---|
| HN2 concentration μg./ml. | Time (hr.) | DNA Adenine | RNA Adenine | Acid-soluble nucleotide adenine* |
| 0 | 1 | 56 | 710 | 14,807 |
| 0 | 2 | 116 | 1,620 | 31,245 |
| 0 | 4 | 265 | 2,800 | 54,270 |
| 20 | 1 | 26 | 358 | 28,985 |
| 20 | 2 | 28 | 724 | 75,660 |
| 20 | 4 | 48 | 1,375 | 140,425 |

* Prior to hydrolysis and chromatography, the acid-soluble fraction was extracted 8 times with *n*-butanol to remove free adenine and adenosine.

Since none of these experiments on ascites tumours gave any indication that HN2 has a specific effect on incorporation of labelled precursors into DNA *in vitro*, an attempt was made to determine whether this was due either to the nature of the ascites tumour or to the limitations inherent in experiments on surviving tissues. For this purpose the effects of HN2 on the DNA and RNA content of growing cultures of Earle's " L " cells were investigated.

A preliminary experiment to determine a suitable mustard concentration showed that the " L " cells are much more sensitive to HN2 than ascites cells. At a concentration of HN2 as low as 1 μg./ml. considerable cell death occurred as shown by the fact that the DNA content of the cultures was reduced to about 65 per cent of the initial value. At concentrations of HN2 of from 0·25 to 0·5 μg./ml. growth continued but the rate of synthesis of nucleic acids was reduced (Table XIII), DNA being affected to a greater extent than RNA so that the ratio of RNA : DNA per culture increased as the mustard concentration was increased.

TABLE XIII.—*Effect of Methyl-di-(2-chloroethyl)amine (HN2) on the Nucleic Acids of Growing Cultures of Earle's " L " Cells*

Figures are given in terms of amount per flask.

| HN2 concentration μg./ml. | DNA μg. | RNA μg. | Ratio RNA/DNA |
|---|---|---|---|
| Zero time control | 50·8 | 64·8 | 1·28 |
| 0 | 128.8 | 137·5 | 1·07 |
| 0·25 | 81·2 | 103·3 | 1·27 |
| 0·33 | 70·2 | 97·5 | 1·39 |
| 0·5 | 57·8 | 91·3 | 1·58 |

The figures given, except the initial control values, are the mean value for 4 replicate cultures.

### DISCUSSION

It is widely believed that mustards have a specific effect on the metabolism of DNA as distinct from RNA, but there is little published evidence to support this point of view. The experiments reported here were carried out to ascertain whether such a specific effect could be demonstrated in mammalian tissues.

No evidence for a specific effect of mustards on DNA synthesis was obtained in experiments with the Ehrlich mouse ascites carcinoma (Tables III–IV, Tables VIII–X) or the MC1M mouse ascites sarcoma (Tables V–VII). Other workers using similar materials have also failed to detect a differential effect of nitrogen mustard on isotope incorporation into DNA and RNA. Adenocarcinoma 755, when treated *in vivo* with HN2, does not show any specific effect of the mustard on the incorporation of $^{32}PO_4^{\equiv}$ into DNA (Davidson and Freeman, 1955). Results of a similar nature have been reported by LePage and Greenlees (1955) for the incorporation of (2–$^{14}$C)glycine into the nucleic acids of ascites cells. Heidelberger and Keller (1955) failed to detect a specific effect of HN2 on the uptake of ($^{14}$C)-formate into the DNA of Flexner-Jobling carcinoma of the rat.

On the other hand, in the present series of experiments some evidence for a specific effect of H and HN2 has been obtained in the intact rabbit and in cells grown in tissue culture. The results obtained from the rabbit experiments (Tables I–II) suggest that, while the mustards depress incorporation of $^{32}PO_4^{\equiv}$ into both DNA and RNA, depression of uptake into DNA is more marked than that into RNA. These results are in agreement with those obtained by Lowrance and Carter (1950) in a similar but less extensive series of experiments with rabbits.

In the tissue culture experiments on the total amount of DNA and RNA present in cultures of control cells and cells treated with concentrations of nitrogen mustard in the range 0·25 to 0·5 $\mu$g./ml. there is a progressive increase in the ratio of RNA : DNA with increasing mustard concentration. This change in the RNA : DNA ratio may be interpreted as evidence that HN2 curtails DNA synthesis to a greater extent than RNA synthesis.

If the biosynthesis of the nucleic acids is considered as being a three-stage process involving synthesis of mononucleotides, their phosphorylation to give nucleoside di- or tri-phosphates, and condensation of the di- or tri-phosphates to form polynucleotides, the evidence so far obtained suggests that HN2 inhibits the process at either the second or third stage. Support for this suggestion may be derived from studies on the effect of mustards on enzymes, *in vitro*. It has been shown that the majority of enzymes are relatively unaffected by the mustards but there is a smaller group of sensitive enzymes consisting mainly of phosphokinases including those necessary for the phosphorylation or dephosphorylation of adenylic residues (Needham, 1948).

With MC1M cells 50 $\mu$g./ml. HN2 depresses $^{32}PO_4^{\equiv}$ incorporation into DNA, nRNA and cRNA (Table V) and also decreases formate incorporation into DNA thymine without producing any clear-cut effect on formate incorporation into nucleic acid purines (Table VI). This means that the inhibition of $^{32}PO_4^{\equiv}$ incorporation is not due to inhibition of purine synthesis. Further evidence for this point of view is found in the experiments showing that neither mouse liver extract (Table VI), AICA riboside (Table X), nor a mixture of nucleosides (Table V) completely reverses the effect of HN2 on incorporation.

In experiments in which the incorporation of labelled formate or adenine into the acid-soluble adenine has been measured, the results show that the specific activities of adenine from the acid-soluble fractions of mustard-treated cells were not lower, and usually much higher, than those of adenine from the acid-soluble fractions of control cells (Tables XI and XII). Thus under conditions in which there is clear-cut evidence of the inhibition of nucleic acid biosynthesis, the synthesis of acid-soluble purine nucleotides is not diminished but enhanced.

These views on the mechanism of action of nitrogen mustard are in agreement with those put forward by Goldthwait (1952) who suggested on the basis of his study of the effect of HN2 on the simultaneous incorporation of ($^{14}C$) formate and (1 : 3–$^{15}N_2$)adenine into the small intestine of the rat, that HN2 did not inhibit the formation of the purine skeleton.

The general conclusion to be drawn from the present studies is that although H and HN2 may exercise a more pronounced inhibitory action on the metabolism of DNA than of RNA, especially in thc intact animal, there is no evidence to show that mustard at any concentration exercises a specific effect on DNA metabolism only.

We should like to thank colleagues at the Ministry of Supply Chemical Defence Experimental Establishment at Porton for helpful discussions, Dr. John Paul of the H.E.R.T. Tissue Culture Laboratory of this Department for valuable help and guidance in the experiments with " L " cells, Dr. G. J. Popjak of Hammersmith Hospital for a gift of mice bearing the Ehrlich ascites carcinoma, Dr. G. Klein of Stockholm for a gift of mice bearing the MC1M sarcoma, and Messrs. Boots Pure Drug Company of Nottingham for a gift of nitrogen mustard.

### SUMMARY

In an attempt to determine whether sulphur and nitrogen mustards exerted a specific effect on DNA metabolism, the effect of H and HN2 on the nucleic acid metabolism of a number of mammalian systems was investigated.

1. In experiments with rabbits *in vivo* mustard exercised a more pronounced inhibitory effect on DNA metabolism than on RNA metabolism. Further evidence for a selective effect on DNA was obtained from experiments with Earle's " L " strain cells grown in tissue culture.

2. No evidence for a differential effect of HN2 on DNA metabolism was obtained from experiments with Ehrlich mouse ascites carcinoma cells incubated, *in vitro*, in ascitic plasma or from MC1M mouse ascites sarcoma cells incubated *in vitro* in ascitic plasma.

3. The evidence obtained suggests that nitrogen mustard depresses nucleic acid synthesis by inhibiting either the phosphorylation of mononucleotides to form di- or tri-phosphates or the condensation of these di- or tri-phosphates to give polynucleotides rather than by inhibiting the biosynthesis of mononucleotides.

### REFERENCES

BODENSTEIN, D. AND KONDRITZER, A. A.—(1948) *J. exp. Zool.*, **107**, 109.

COHEN, P. P.—(1951) 'Manometric Techniques and Tissue Metabolism', edited by W. W. Umbreit, R. H. Burris and J. J. Stauffer. 2nd ed. Minneapolis (Burgess Publishing Co.), p. 193.

DAVIDSON, J. D. AND FREEMAN, B. F.—(1955) *Cancer Res., Suppl.*, **3**, 97.

Goldthwait, D. A.—(1952) *Proc. Soc. exp. Biol. N.Y.*, **80**, 503.
Grunberg-Manago, M., Oritz, P. J. and Ochoa, S.—(1956) *Biochim. biophys. Acta*, **20**, 269.
Hanks, J. H. and Wallace, R. E.—(1949) *Proc. Soc. exp. Biol. N.Y.*, **71**, 196.
Heidelberger, C. and Keller, R. A.—(1955) *Cancer Res. Suppl.*, **3**, 106.
Herriott, R. M.—(1950–51) *J. gen. Physiol.*, **34**, 761.
Klein, G.—(1951) *Exp. Cell Res.*, **2**, 518.
Kornberg, A.—(1957) 'The Chemical Basis of Heredity', edited by W. D. McElroy and B. Glass. Baltimore (Johns Hopkins Press), p. 579.
LePage, G. A. and Greenlees, J. L.—(1955) *Cancer Res. Suppl.*, **3**, 102.
Lowrance, P. B. and Carter, C. E.—(1950) *J. cell comp. Physiol*, **35**, 387.
Needham, D. M.—(1948) *Biochem. Soc. Symp.*, *No.* 2, p. 16.
Paul, J.—(1958) *Analyst* (in Press).
Philips, F. S.—(1950) *Pharmacol. Rev.*, **2**, 281.
Sanford, K. K., Earle, W. R. and Likely, G. D.—(1948) *J. nat. Cancer Inst.*, **9**, 229.
Schulman, M. P., Sonne, J. C. and Buchanan, J. M.—(1952) *J. biol. Chem.*, **196**, 499.
Smellie, R. M. S., Humphrey, G. F., Kay, E. R. M. and Davidson, J. N.—(1955) *Biochem. J.*, **60**, 177.
*Idem*, Thomson, R. Y. and Davidson, J. N.—(1958) *Biochem. biophys. Acta* (in Press).
*Idem*, Thomson, R. Y., Goutier, R. and Davidson, J. N.—(1956) *Biochim. biophys. Acta*, **22**, 585.
Thomson, R. Y., Smellie, R. M. S. and Harrington, H.—(1957) *Biochem. J.*, **67**, 17.